# Magnetic Resonance Imaging Pilot Study of Intravenous Glyburide in Traumatic Brain Injury

**DOI:** 10.1089/neu.2019.6538

**Published:** 2019-12-11

**Authors:** Howard M. Eisenberg, Martha E. Shenton, Ofer Pasternak, J. Marc Simard, David O. Okonkwo, Christina Aldrich, Feng He, Sonia Jain, Erik G. Hayman

**Affiliations:** ^1^ Department of Neurosurgery, University of Maryland School of Medicine, Baltimore, Maryland.; ^2^ Departments of Psychiatry and Radiology, Brigham and Women's Hospital, Harvard Medical School, Boston, Massachusetts.; ^3^ Department of Research and Development, VA Boston Healthcare System, Brockton Division, Brockton, Massachusetts.; ^4^ Department of Neurological Surgery, University of Pittsburgh School of Medicine, Pittsburgh, Pennsylvania.; ^5^ Department of Family Medicine and Public Health, University of California San Diego, La Jolla, California.

**Keywords:** contusion, edema, glyburide, MRI, SUR1, TBI

## Abstract

Pre-clinical studies of traumatic brain injury (TBI) show that glyburide reduces edema and hemorrhagic progression of contusions. We conducted a small Phase II, three-institution, randomized placebo-controlled trial of subjects with TBI to assess the safety and efficacy of intravenous (IV) glyburide. Twenty-eight subjects were randomized and underwent a 72-h infusion of IV glyburide or placebo, beginning within 10 h of trauma. Of the 28 subjects, 25 had Glasgow Coma Scale (GCS) scores of 6–10, and 14 had contusions. There were no differences in adverse events (AEs) or severe adverse events (ASEs) between groups. The magnetic resonance imaging (MRI) percent change at 72–168 h from screening/baseline was compared between the glyburide and placebo groups. Analysis of contusions (7 per group) showed that lesion volumes (hemorrhage plus edema) increased 1036% with placebo versus 136% with glyburide (*p* = 0.15), and that hemorrhage volumes increased 11.6% with placebo but decreased 29.6% with glyburide (*p* = 0.62). Three diffusion MRI measures of edema were quantified: mean diffusivity (MD), free water (FW), and tissue MD (MDt), corresponding to overall, extracellular, and intracellular water, respectively. The percent change with time for each measure was compared in lesions (*n* = 14) versus uninjured white matter (*n* = 24) in subjects receiving placebo (*n* = 20) or glyburide (*n* = 18). For placebo, the percent change in lesions for all three measures was significantly different compared with uninjured white matter (analysis of variance [ANOVA], *p* < 0.02), consistent with worsening of edema in untreated contusions. In contrast, for glyburide, the percent change in lesions for all three measures was not significantly different compared with uninjured white matter. Further study of IV glyburide in contusion TBI is warranted.

## Introduction

More than 2 million Americans suffer injury or death due to traumatic brain injury (TBI) each year, with TBI being a major contributing factor in over one-third of trauma-related deaths.^1^ A significant proportion of brain trauma survivors experience long-term disability, resulting in large societal costs from both direct care as well as from loss of productivity by previously healthy individuals. However, despite decades of research, pharmacological therapy for TBI remains confined to early seizure prophylaxis and, possibly, barbiturates, based on a small trial showing better intracranial pressure (ICP) control compared with placebo.^2^ More than 30 controlled clinical trials of pharmacological therapies, however, have failed to improve mortality or outcome following TBI.^3^

TBI proceeds in two phases, an immediate primary injury to the brain due to structural disruption at the time of impact, and delayed, progressive injury that results from a variety of pathophysiological processes collectively termed secondary injury, including increasing edema and hemorrhagic progression of contusion.^4–6^ Recent research indicates a previously undescribed basis for some forms of post-traumatic secondary injury. Specifically, experimental models of contusion TBI,^7–9^ as well as patient-derived clinical samples,^9,10^ consistently demonstrate both upregulation and pathological pairing of sulfonylurea receptor 1 (SUR1) with the non-selective cation channel, transient receptor potential melastatin 4 (TRPM4), to form SUR1-TRPM4 channels in brain endothelium, astrocytes, and macrophages following contusion TBI.

Several pre-clinical studies provide a rationale for targeting SUR1 following contusion TBI. Inhibition of SUR1 using glyburide or gene suppression reduces hemorrhagic progression and edema, and may be associated with functional improvements after contusion TBI, findings confirmed by several independent research groups.^7–9,11^ Similarly, glyburide reduces hemorrhagic progression after contusion injury to the spinal cord.^12,13^ Further, in patients with TBI, cerebrospinal fluid (CSF) levels of SUR1 may act as a biomarker that predicts swelling and outcome.^14^ Also, genetic studies of SUR1 and TRPM4 polymorphisms in TBI patients have identified several SUR1 and TRPM4 variants linked to brain swelling or cerebral edema.^15–17^

Glyburide, a member of the sulfonylurea class of oral anti-diabetic drugs, is a potent pharmacological inhibitor of SUR1-TRPM4 channels.^18^ Recent clinical experience with intravenous (IV) glyburide in the treatment of large hemispheric infarction indicates that glyburide is well tolerated, without clinically significant off-target effects aside from occasional but manageable hypoglycemia.^19,20^ Clinical experience with glyburide treatment for TBI is limited to two small randomized controlled trials that used the oral form of the drug, and showed a probable benefit of glyburide with regard to contusion expansion and neurological outcome.^21,22^

Here, we undertook a small Phase II multi-institutional randomized controlled trial to evaluate the safety and mitigation of hemorrhagic progression and edema with IV glyburide following TBI. Surrogate biomarkers derived from magnetic resonance imaging (MRI) were the primary outcome measures.

## Methods

### Study organization

This was a double-blind, randomized, placebo-controlled, Phase II trial of IV glyburide in patients with TBI (ClinicalTrials.gov identifier: NCT01454154). It was one of several clinical trials within the Department of Defense INTRuST [Injury &Traumatic Stress], Post-Traumatic Stress Disorder - Traumatic Brain Injury Clinical Consortium, but was the only consortium study focused on acute TBI. UCSD (University of California, San Diego) was the INTRuST coordinating center, providing the informatics and biostatistic scores. Remedy Pharmaceuticals, Inc. provided the drug (IV glyburide; a.k.a. RP-1127 or BIIB-093) and placebo (excipients) for the study, under an Investigational New Drug Application from the U.S. Food and Drug Administration. The study was supervised by the Institutional Review Boards of the individual centers, UCSD, and the USAMRMC (U.S. Army Medical Research and Materiel Command).

Analysis of the MRI data was conducted by the Psychiatry Neuroimaging Laboratory at Brigham and Women's Hospital, Harvard Medical School, Department of Psychiatry, which was the core imaging center for the INTRuST consortium. The lead clinical center for this trial was UMSOM (University of Maryland School of Medicine), which later was joined by two other centers, UCSD and UPMC (University of Pittsburgh Medical Center), although ultimately, 76% of the total randomized participants were enrolled at UMSOM. The primary biostatistician (SJ) had full access to all the clinical data in the study. The project principal investigator (HME) had final responsibility for the decision to submit for publication.

### Subject enrollment and randomization

The study protocol called for enrolling 100 participants into one of two arms, IV glyburide or placebo, at a 1:1 ratio. However, the coordinating center reduced the number of participating sites from five to three, and the study was terminated after 29 subjects had been randomized, 30 months after starting, due to slow enrollment and completion of the allotted period of funding. All participants' legally authorized representatives (LARs) provided written informed consent at enrollment. Subjects were consented and enrolled into the study, but were not randomized until completion of the T1-weighted (T1w) and diffusion tensor imaging (DTI) sequences of the baseline MRI. Randomization and infusion of drug or placebo then proceeded, as long as infusion could be started within 10 h of the trauma. Participants were randomly assigned to receive IV glyburide or placebo in a 1:1 ratio from a centralized, web-based randomization system (Interactive Web Randomization System). At randomization, subjects were stratified on the basis of their Glasgow Coma Scale (GCS) score (4–8 and 9–14) obtained free of the effects of sedating and/or paralytic drugs, closest to the time of randomization.

The major inclusion/exclusion criteria were age (18–75 years), GCS score 4–14, start of infusion within the 10-h window, history of treatment with sulfonylurea drugs, and perceived inability to tolerate the initial MRI scan (full inclusion/exclusion criteria are provided in Supplementary Appendix S1). The INTRuST coordinating center (except for the Biostatistics Core), Remedy Pharmaceuticals, the principal investigator, site investigators, patients, imaging core, and outcomes personnel were blinded to treatment.

### Treatments

The bolus and the infusion concentrations of glyburide were both 5.3 μg/mL. Glyburide was infused IV as a loading dose, 0.13 mg over 2 min, then 0.16 mg/h for 6 h, and then 0.11 mg/h for 66 h (total daily dose on day 1 was 3.12 mg and on days 2 and 3 was 2.67 mg/day) versus placebo (similar in appearance to drug and at the same rate). This dose was based on data from a Phase I drug escalation safety study.^23^ The infusion protocol and 10-h window were identical to that used in a Phase II trial of glyburide in large hemispheric infarction.^20^ Drug vials, preparation bags, and tubing were identical in appearance for both treatment groups. Concomitant treatments followed national practice guidelines for TBI patients.^24^

### Study outcome measures

The pre-specified primary efficacy objective was to assess whether subjects treated with IV glyburide would show a decrease in MRI-defined edema and/or hemorrhage, compared with placebo-treated patients. The study protocol did not specify which specific measure of edema and/or hemorrhage would be primary (see below for the measures that were quantified). Efficacy outcome was assessed using data from two sequential MRIs, a baseline MRI before infusion of drug or placebo, and another MRI after completion of infusion, specifically indexing water and blood. The interval between the baseline MR scan and the second scan was initially specified as 72 ± 12 h. However, very early in the study, we realized that this was not possible in some subjects, when the second scan had to be delayed mainly due to ICP elevations above guideline levels when participants were positioned flat for scanning. The protocol then was revised to extend the interval to 168 h (7 days). The number of patients scanned within the 72-h window, the 168-h window, and beyond 168 h are reported separately.

A pre-specified secondary efficacy outcome was the Glasgow Outcome Scale Extended (GOS-E) at 90 days.

The primary safety objective was to assess the safety (the incidence of mortality, adverse events [AEs], and serious adverse events [SAEs]) and tolerability of IV glyburide compared with placebo in subjects with severe, moderate, or complicated mild TBI.

### MRI data acquisition

MRI scans were acquired at each site on a 1.5T scanner. At UMSOM, two identical Siemens Avanto scanners were used (baseline and follow-up scans for each subject were always on the same scanner). At the UCSD and UPMC sites, a single 1.5 Signa GE scanner was used. One single phantom was used to standardize all scanners at all sites. Also, at each site, patients were always scanned using the same scanner.

The MRI acquisition was set to minimize differences between vendors and included anatomical sequences: high resolution (1 × 1 × 1 mm^3^) T1w (Siemens: magnetization-prepared rapid gradient-echo [MPRAGE], inversion time of 1100 msec, flip angle 7 degrees; GE: spoiled gradient recalled [SPGR], inversion time of 600 msec, flip angle 10 degrees], high resolution T2, proton density (Dual echo, echo time [TE]: 12 msec and 100 msec; 1 × 1 × 1 mm^3^), and a clinical fluid-attenuated inversion recovery (FLAIR) sequence (1.3 mm × 1 mm × 5 mm; inversion time: 2500 msec). Two additional scans of interest were diffusion MRI and susceptibility-weighted imaging (SWI). The diffusion MRI scan included a multi-shell design with 46 unique gradient orientations and a total of 66 volumes spread over 5 nested b-shells of 1 × 0, 3 × 50, 6 × 250, 10 × 500, 30 × 900, and 16 × 1400 sec/mm^2^, and the following parameters: 2.5 mm isotropic, 66 slices (Siemens: repetition time [TR]/TE 8500/85 msec; GE: TR/TE 9550 msec/min). The SWI scan was a 3D gradient-echo scan (1 mm × 1 mm × 1.5 mm, TR/TE 50/40 msec, flip angle: 15 degrees) outputting the magnitude and phase images.

### Regions of interest

**FIG. 1.**
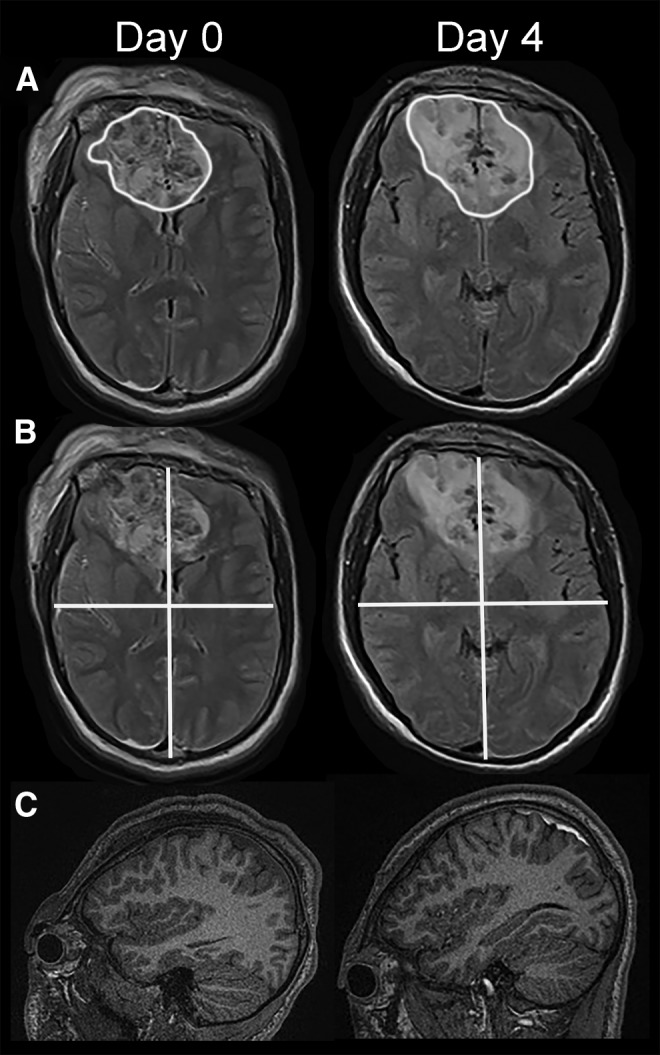
Representative FLAIR and T1w images illustrating ROIs that were studied. **(A,B)** Axial slices of FLAIR images on day 0 and day 4 showing lesion ROIs (A) and quadrant ROIs (B); to compensate for different gantry angles on different days, the slices illustrated are the first full slice above the right orbital roof**. (C)** Sagittal slices of T1w images on day 0 and day 4 showing uninjured white matter. All images are from patient 14, who received IV glyburide. FLAIR, fluid-attenuated inversion recovery; IV, intravenous; ROI, region of interest; T1w, T1-weighted.

**FIG. 2.**
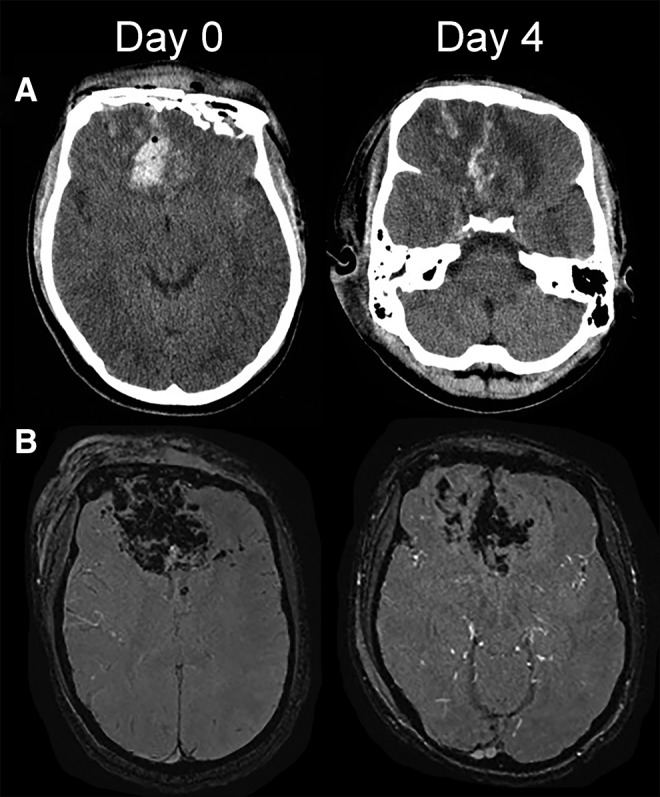
Representative CT and SW images illustrating hemorrhages. **(A,B)** Axial slices of CT (A) and SW (B) images on day 0 and day 4 showing hemorrhages; to compensate for different gantry angles on different days, the slices illustrated are the first full slice above the right orbital roof. All images are from patient 14, who received IV glyburide. CT, computed tomography; IV, intravenous; SW, susceptibility-weighted.

Three regions of interest (ROIs) were analyzed for volumetric changes: the total lesion volume (blood plus edema) (*n* = 15), blood alone (*n* = 14), and the total brain volume (*n* = 24). Four additional ROIs were analyzed for changes in edema: uninjured white matter (*n* = 24); lesions, that is, contusions (*n* = 14); all quadrants in a coronal slice (four per subject; *n* = 96); and the maximally affected quadrant (one per subject; *n* = 24). For lesion ROIs (blood plus edema), we aligned images from computed tomography (CT) and MRI. Lesion volumes were calculated by electronically tracing the areas of the lesion on sequential slices. Edema was identified predominantly based on hyperintensities in the FLAIR and T2 sequences (Fig. 1A). Hemorrhage was identified based on hypointensities in the SWI contrast (following phase enhancement^25^) and blood densities on CT (Fig. 2A,B). The final lesion ROIs were reviewed and modified by a neurosurgeon, a neuroradiologist, and a researcher specializing in image analysis, blinded to treatment arm. Lesions were excluded if they were outside brain (e.g., subarachnoid and extra-axial and interventricular hemorrhages were excluded). Apparent lesions also were excluded if they were directly related to a treatment that took place between the two MRIs (e.g., surgery or insertion of an intraventricular catheter).

For uninjured white matter ROIs, which were intended as an exploratory approach to identify global effects of the drug, we used expectation-maximization (EM) segmentation of the T1w image (FAST; FSL) (Fig. 1C). The white matter segment was eroded by one voxel to avoid partial volume voxels, and we excluded all lesions that were defined in the previous approach (the lesion segments here were first dilated by one voxel).

For all quadrant ROIs, which also were intended as an exploratory approach to identify global effects of the drug, each quadrant within a coronal brain slice was studied separately, whether or not a lesion was present (Fig. 1B).

For the maximally affected quadrant, the single quadrant of a coronal brain slice with the maximal change in edema parameters for each subject was used.

### MRI data analysis

All evaluators and those involved in management of subjects including the PI (HME) were blinded to treatment arm and other clinical or demographic information. All imaging data were subjected to visual quality control to exclude images with severe motion or other artifacts. The diffusion data were manually masked to exclude non-brain areas. The anatomical images were co-registered using rigid transformations (within time-points) and affine transformations (between time-points). In addition, the anatomical images were manually masked based on the T1w image, all by investigators blind to treatment arm.

The diffusion MRI data were pre-processed using motion and eddy correction applied by means of affine transformations (FLIRT; FSL). The aligned images then were analyzed using the DTI model,^26,27^ and the free-water imaging model,^28,29^ yielding voxel-wise maps of DTI measures and free-water imaging measures.

Three MRI diffusion measures were used to assess edema: (1) mean diffusivity (MD); (2) the volume fraction of free water (FW); and (3) tissue mean diffusivity (MDt). MD was derived from the DTI, whereas FW and MDt were derived from the free-water imaging model. MD provides an overall measure of diffusivity; as water molecules move faster on average, the MD measure is higher. FW and MDt measures further deconstruct the signal contribution into extracellular (FW) and intracellular (MDt) water. The free-water model separates the signal into water molecules that are free to diffuse in the extracellular space (i.e., diffusing at 3 × 10^–3^ mm^2^/sec), and all remaining molecules that are restricted or hindered by membranes and other tissue-related obstacles.

Higher fractions of extracellular space, which are expected in vasogenic edema, would increase FW. Changes in the geometry, and hence the speed of water molecules in proximity to tissue, would affect the MDt, whereas less restricted motion would increase MDt. On the other hand, cellular swelling (i.e., cytotoxic edema) would decrease the non-restricted space, decreasing FW, and modifying MDt. Values for MD, FW, and MDt were averaged for each of the four ROIs described above. Because edema was an important end-point due to the expected drug mechanism, and because the ROIs were large, we did not consider anisotropic measures such as fractional anisotropy or radial or axial diffusivities. Hemorrhage was assessed by the number of positive voxels on SWI within the boundaries of the lesion ROIs.

### Sample size calculation

A sample size calculation was performed based on a two-sided, two-sample *t* test to compare published differences in absolute values in apparent diffusion coefficient (ADC) in TBI patients relative to normal subjects, and was computed using standardized effect sizes. With an anticipated sample size of 100 participants (equal allocation between active and placebo arms), the study would have 80% power to detect a 60% standardized change in treatment arms, assuming 10% attrition over 72 h and α = 0.05.

### Statistical analysis plan

Analyses were to incorporate the intent-to-treat principle, namely, all randomized participants would be included in the analysis according to their treatment assigned at randomization. The safety analysis was to be performed on the safety population only, that is, all those who were exposed to any study drug. No adjustments for multiple comparisons were to be made for secondary analyses, and a *p*-value of 0.05 was be considered statistically significant. The final statistical analysis plan was to be determined by the INTRuST Biostatistics Core within 6 months before study end.

### Statistical analysis

All MRI measures were evaluated as the “percent change” of the second MRI compared with the screening/baseline MRI, before infusion of study drug or placebo. Summary measures (mean, standard deviation, median, first and third quartiles, minimum and maximum values) described the data overall and by study arm for each outcome at screening/baseline and at the second scan. Summary measures were produced overall and by study arm for each outcome for the percent change from screening/baseline to the second scan. The primary analysis was based on a two-sided, two-sample Wilcoxon rank sum test to compare the glyburide and placebo arms. A secondary analysis of the three MRI measures of edema (MD, FW and MDt) was based on an analysis of variance (ANOVA) to compare four groups: lesions versus uninjured white matter, in the glyburide and placebo arms. Safety data were summarized overall and by treatment groups. Fisher's exact test was used to compare the number of subjects between groups who experienced any adverse events (AEs). Statistical analyses were performed in R version 3.1.1. (www.r-project.org) or GraphPad Prism version 8.

## Results

### General

In all, 1483 potentially qualified participants were screened at the three centers, the largest number at UMSOM (924). Of those screened, 38 were consented. The most frequent reasons not to consent included: GCS score out of range (529), perceived inadequate time from admission to hospital to start of infusion (202), and LAR not available (95). Other less common reasons included an anticoagulant medication and elevated blood alcohol (see Supplementary Fig. S1). Of the 38 consented, 9 could not be randomized (5 could not complete the baseline MRI and 4 could not start the infusion on time). Of the 29 subjects randomized (15 in the glyburide group, 14 in the placebo group), 1 randomized to the IV glyburide arm did not receive the drug because of withdrawal of consent. Two subjects were randomized or infused incorrectly. Errors included randomization or infusion outside of the time window, and protocol-unspecified stopping and restarting of infusion. These protocol violations occurred early in the trial. These subjects were included in the efficacy and safety analysis, due to the intent-to-treat design. However, data from these subjects were not included when reporting details about infusion and completing infusion according to protocol. Thus, 28 participants completed the acute phase of the study and reached their primary end-points. All 26 uncensored participants had the 72-h infusion of the study drug beginning within 10 h of injury.

### Baseline characteristics

Seventy-two percent of the randomized participants were male, 10 in the placebo arm and 11 in the glyburide arm, and 28% were female (4 in each arm). The median age in the placebo arm was 23 years (66 maximum) and it was 22 years in the glyburide arm (60 maximum). ICP was monitored at randomization in 20 participants. The median ICP at randomization was 14 mm Hg in the placebo arm (maximum 19) and 11 mm Hg in the glyburide arm (maximum 16). Randomization median P_a_O_2_ and mean arterial pressure were virtually identical in the two arms and were within normal ranges. Randomization median blood glucose was 138 mg/dL (185 maximum) in the placebo arm and 139mg/dL (maximum 248) in the glyburide arm.

GCS scores obtained at the time closest to randomization are shown in Table 1. No patient with a GCS score >11 met requirements for randomization. Twenty-five of the 29 randomized participants had GCS scores 6–10, 86% of the cohort. A GCS score of 7 was the most common score. In this regard, the cohort was in the range of lower-moderate to upper-severe injury.

**Table 1.** Glasgow Coma Scale Score at Randomization

GSC, Glasgow Coma Scale.

### Laboratory findings

Routine blood chemistries were collected at screening, baseline, and at days 1, 2, 3, and 4. There were no group differences with regard to most of these measures. Glucose was monitored; blood samples were taken hourly for the first 24 h, then every 4 h for the next 24 h. Hypoglycemia was managed by a detailed protocol (see Supplementary Appendix S2), which specified glucose levels, and specified administering glucose and, if necessary, reducing or suspending the infusion of the study drug (investigator blind to treatment arm). Two participants had glucose levels <55 mg/dL at one time-point. None of the 26 uncensored subjects had suspension of drug infusion; all cases of hypoglycemia were managed by infusion of glucose. The glucose data were analyzed using an area under the curve (AUC) approach by 24 h. No significant difference between groups was found. Hourly collection of vital signs, which included blood pressure and heart rate, were also analyzed using AUC by 24 h, with no significant differences found between groups.

### Adverse events

There were no deaths. Three patients (2 in the glyburide arm, 1 in the placebo arm) had transient neurological worsening (decreased GCS score of 2 points in 2 consecutive h and/or a newly dilated pupil). There were no significant differences in the frequencies of defined AEs between the two treatment groups (glyburide, 87% vs. placebo, 100%; *p* = 0.48), nor was there a significant difference with regard to severe adverse events (SAEs) (glyburide, 20% vs. placebo, 14%; *p* = 0.99). Overall, there were 7 SAEs in 5 participants (Table 2).

**Table 2.** Serious Adverse Events (SAEs) by Treatment Arm

	Glyburide	Placebo	Total
Not related	3 (75%)	3 (100%)	6 (85.7%)
Unlikely	0 (0%)	0 (0%)	0 (0%)
Possible	0 (0%)	0 (0%)	0 (0%)
Probable	1 (25%)	0 (0%)	1 (14.3%)
Definite	0 (0%)	0 (0%)	0 (0%)
Total	4 (100%)	3 (100%)	7 (100%)

### MRI analysis—general

Of the 26 uncensored participants, all had a 72-h drug infusion beginning within 10 h of trauma. Of the 28 participants randomized and infused, 17 had their second scan within the initially proposed 72 ± 12-h window (drug 8, placebo 9), whereas 7 additional participants had their second scan at or before 168 h (drug 3, placebo 4). Four others did not have a second scan within the 168-h window. For the 14 participants with identified lesions (contusions), 10 participants had their second scan within the 72 ± 12-h interval (drug 5, placebo 5). The other 4 had their second scan at or before 168 h (drug 2, placebo 2). The distribution of randomization GCS for the lesion ROI cohort is shown in Table 3.

**Table 3.** Randomization GCS for Subjects with Lesions (contusions)

GCS	Glyburide	Placebo	Total
5	1 (14.3%)	0 (0%)	1 (7.1%)
6	1 (14.3%)	1 (14.3%)	2 (14.3%)
7	3 (42.9%)	2 (28.6%)	5 (35.7%)
8	0 (0%)	1 (14.3%)	1 (7.1%)
9	1 (14.3%)	1 (14.3%)	2 (14.3%)
10	1 (14.3%)	2 (28.6%)	3 (21.4%)
Total	7 (100%)	7 (100%)	14 (100%)

GCS, Glasgow Coma Scale; ROI, region of interest.

### MRI analysis—volumetric changes

Three ROIs were analyzed for volumetric changes: the total lesion volume (blood plus edema), the volume of blood alone, and the total brain volume (Table 4). Lesion volumes increased in both treatment arms, but more so in the placebo arm. The volume of blood in lesions increased in the placebo arm, whereas it decreased in the glyburide arm (Fig. 2A,B). Neither of these differences reached statistical significance. Changes in total brain volume were small.

**Table 4.** Percent Changes in Three Volumes That Were Analyzed

Volume		Mean	SD	N	P
Total lesion (blood + edema)	G	136.06	195.62	7	0.15
	P	1036.29	1963.28	8	
Blood alone	G	–29.55	36.75	7	0.62
	P	11.64	91.37	7	
Total brain	G	2.01	9.02	11	0.13
	P	2.16	3.26	13	

G, glyburide; P, placebo; SD, standard deviation.

### MRI analysis—measures of edema in four ROIs

The percent change from screening/baseline to the second scan was analyzed for three measures of edema: MD (overall water), FW (extracellular water) and MDt (intracellular water); these three measures of edema were evaluated in four ROIs: uninjured white matter, lesions, all quadrants, and the maximally affected quadrant in each subject (Table 5). Overall, the greatest differences between treatment arms was found in lesion ROIs, in which all three measures of edema showed larger percent increases with placebo compared with glyburide. In pairwise comparisons, only MDt in the all-quadrants ROI reached statistical significance between glyburide and placebo.

**Table 5.** Percent Changes in Three MRI Measures of Edema in Four Regions of Interest

Region of interest	Measure of edema	Treatment	Mean	SD	N	P
Uninjured white matter	MD	G	3.95	6.40	11	0.49
		P	1.55	3.14	13	
	FW	G	11.74	17.36	11	0.28
		P	3.24	7.84	13	
	MDt	G	1.70	3.66	11	0.84
		P	0.93	2.05	13	
Lesions	MD	G	6.27	9.21	7	0.26
		P	22.04	23.94	7	
	FW	G	10.73	13.99	7	0.32
		P	44.96	57.68	7	
	MDt	G	2.41	6.67	7	0.21
		P	9.66	8.31	7	
All quadrants	MD	G	3.20	5.94	44	0.48
		P	3.71	5.13	52	
	FW	G	12.44	15.25	44	0.42
		P	10.20	15.80	52	
	MDt	G	0.68	3.10	44	0.05
		P	1.33	2.04	52	
Maximally affected quadrant	MD	G	6.37	6.96	11	0.95
		P	6.81	7.27	13	
	FW	G	20.29	16.95	11	0.49
		P	16.98	22.69	13	
	MDt	G	2.36	3.53	11	0.36
		P	2.90	2.33	13	

FW, free water; G, glyburide; MD, mean diffusivity; MDt, tissue mean diffusivity; MRI, magnetic resonance imaging; P, placebo; SD, standard deviation.

**FIG. 3.**
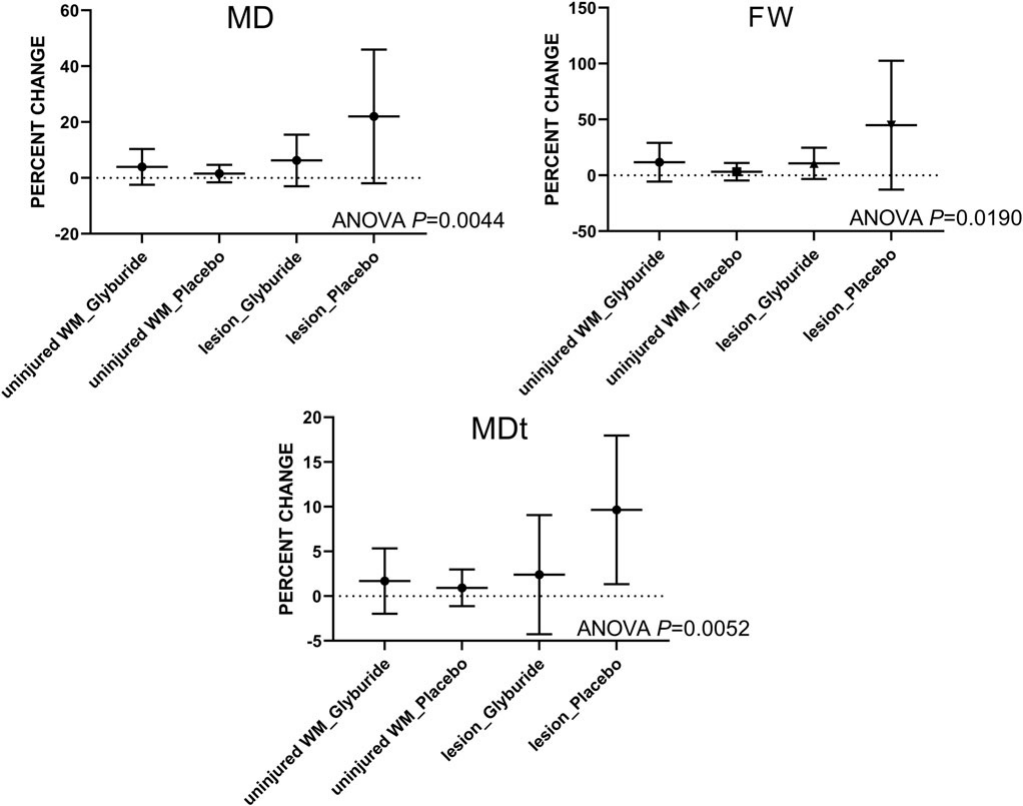
Measures of edema in lesions versus uninjured white matter. Percent changes in MD, FW, and MDt were compared in uninjured WM and in lesions for the two treatment arms. All three measures of edema showed a significant difference (ANOVA, *p* < 0.02). For each measure of edema, comparing the two placebo arms, there was a significant difference between uninjured WM and lesions, whereas comparing the two glyburide arms, there was no significant difference between uninjured WM and lesions. ANOVA, analysis of variance; FW, free water; MD, mean diffusivity; MDt, tissue mean diffusivity; WM, white matter.

We performed a secondary analysis of the three measures of edema, comparing changes in uninjured white matter and in lesions in the two treatment arms (Fig. 3). ANOVA showed a significant difference in all three measures of edema (*p* < 0.02). Post hoc comparisons showed no significant effect of drug in uninjured white matter or in lesions, as in the primary pairwise analyses. However, comparing the placebo arms, there was a significant difference between uninjured white matter and lesions in all three measures of edema, consistent with worsening of edema with time in untreated contusions. By contrast, comparing the glyburide arms, there was no significant difference between uninjured white matter and lesions for any of the three measures of edema.

### Clinical outcomes

As judged by the GOS-E at 90 days, half of the patients in the glyburide group made a good recovery, although 3 were categorized as either vegetative or severely disabled (Table 6). Dichotomizing for GOS-E ≤4 versus >4 at 90 days showed no significant difference between groups (*p* = 0.32).

**Table 6.** GOS-E at 90 Days

	Glyburide	Placebo
Dead	0 (0%)	0 (0%)
Vegetative state (VS)	2 (16.67%)	0 (0%)
Lower severe disability (Lower SD)	1 (8.33%)	0 (0%)
Upper severe disability	0 (0%)	1 (7.69%)
Lower moderate disability (Lower MD)	2 (16.67%)	1 (7.69%)
Upper moderate disability (Upper MD)	1 (8.33%)	7 (53.85%)
Lower good recovery (Lower GR)	3 (25%)	4 (30.77%)
Upper good recovery (Upper GR)	3 (25%)	0 (0%)

GOS-E, Glasgow Outcome Scale Extended.

## Discussion

We sought to assess two principal aspects of glyburide therapy in TBI, namely whether glyburide can be safely administered early after moderate-to-severe TBI, and whether glyburide ameliorates secondary injury, specifically, the development of edema and the delayed expansion of hemorrhage.

The severity of injury in the subjects reported here—moderate (GCS 9–12) and the better end of severe (GCS 6–8) injury, with a few sustaining severe polytrauma—was likely influenced by multiple factors, including the time for transfer from the scene, resuscitation, obtaining consent from a LAR, and the requirement for early MRI, which together caused many screened patients not to be randomized. No patient with GCS >11 met requirements for randomization, which may reflect the fact that patients with lesser injuries are unlikely to be transferred to the R. Adams Cowley Shock Trauma Center at the University of Maryland, where most patients were recruited.

The cohorts, glyburide and placebo, reported here were small, but they were well balanced with regard to important predictors, including randomization GCS, age, and ICP in the overall, as well as in the lesion ROI subset. They also were balanced as to the time the second MRI was acquired, at 72 h or later. There were no important differences in the baseline physiological data of blood pressure, glucose, or ICP as analyzed by AUC.

No safety concerns were reported. Only two subjects required correction of asymptomatic hypoglycemia by infusion of glucose, and drug infusion did not have to be stopped in any subject. With TBI, hypoglycemia may be detrimental to neurological recovery due to increased demands of brain energy metabolism.^30^ However, patients treated with glyburide demonstrated glucose levels comparable to those of controls. Studies of IV glyburide in ischemic stroke^19,20^ have found a similar absence of symptomatic hypoglycemia. This small trial indicates that early use of IV glyburide in moderate-to-severe TBI may be safe, provided that blood glucose is closely monitored and properly managed.

MRI parameters for edema and blood indicated that glyburide may be useful in contusion TBI. Statistical significance was elusive in pairwise comparisons, likely due to the small cohorts, especially with contusions (7 per group), combined with the large variance that is typical in studies of TBI. Nevertheless, interesting observations were made. The increase in lesion volume (hemorrhage plus edema) over time was many-fold greater in placebo- compared with glyburide-treated subjects, and the blood volume increased in placebo-treated subjects, whereas it decreased in glyburide-treated subjects. Notably, an analysis comparing uninjured white matter versus lesions showed that in placebo-treated subjects, measures of edema increased significantly with time, whereas they did not in glyburide-treated subjects. Together, our findings provide preliminary evidence of a favorable effect of glyburide in human TBI that mirrors pre-clinical findings.^7–9^ The absence of a significant effect on clinical outcome was not unexpected, given the small sample size and the potential for multiple confounders.

Given the pre-clinical evidence showing an effect of glyburide on hemorrhagic progression of contusion,^7–9^ we hypothesized that glyburide administration would reduce hemorrhagic expansion. In the literature on contusion TBI, hemorrhagic expansion is observed in ∼50% of cases^5,6^ whereas in this small study, no subjects exhibited frank hemorrhagic expansion. Importantly, albeit unexpectedly, glyburide-treated subjects demonstrated a marked decrease in hemorrhage volume, whereas a small increase was observed with placebo. The observed decrease in hemorrhage volume is not readily accounted for solely by a reduction in expansion. Although speculative, the observed reduction may derive from an effect of glyburide on macrophages, which play a key role in hemorrhage resorption.^31–33^ Macrophages that are present within hemorrhagic contusions are known to upregulate SUR1-TRPM4,^9^ and macrophage phagocytic activity is reportedly enhanced by glyburide.^34,35^

There are several limitations of this trial. First, the trial included only 28 participants. Second, the study population was heterogeneous, in that only half (14/28) had contusions, the type of injury in which an effect of glyburide may be the most anticipated. Third, the timing of follow-up MRI was heterogeneous, potentially limiting the interpretation of the effects of IV glyburide. Fourth, the drug was started nearly 10 h after trauma. In light of the time and efficacy interaction of glyburide in pre-clinical TBI studies,^7^ this comparatively late administration may have attenuated the observed effect size. Fifth and finally, it is possible that a higher dose of IV glyburide than that used in this study might have led to a more robust clinical effect. Further study is needed to confirm the results reported here that glyburide may ameliorate both edema and hemorrhagic progression in moderate-to-severe contusion TBI.

## Supplementary Material

Supplemental data
