# Supplementary material for: Magnetic Resonance Imaging Pilot Study of Intravenous Glyburide in Traumatic Brain Injury
Source: J Neurotrauma. 2019 Dec 11;37(1):185–93. doi: 10.1089/neu.2019.6538 (PMC6921286; doi:10.1089/neu.2019.6538)
Supplement: Supplemental data [file Supp_Data.pdf]

Supplementary Data

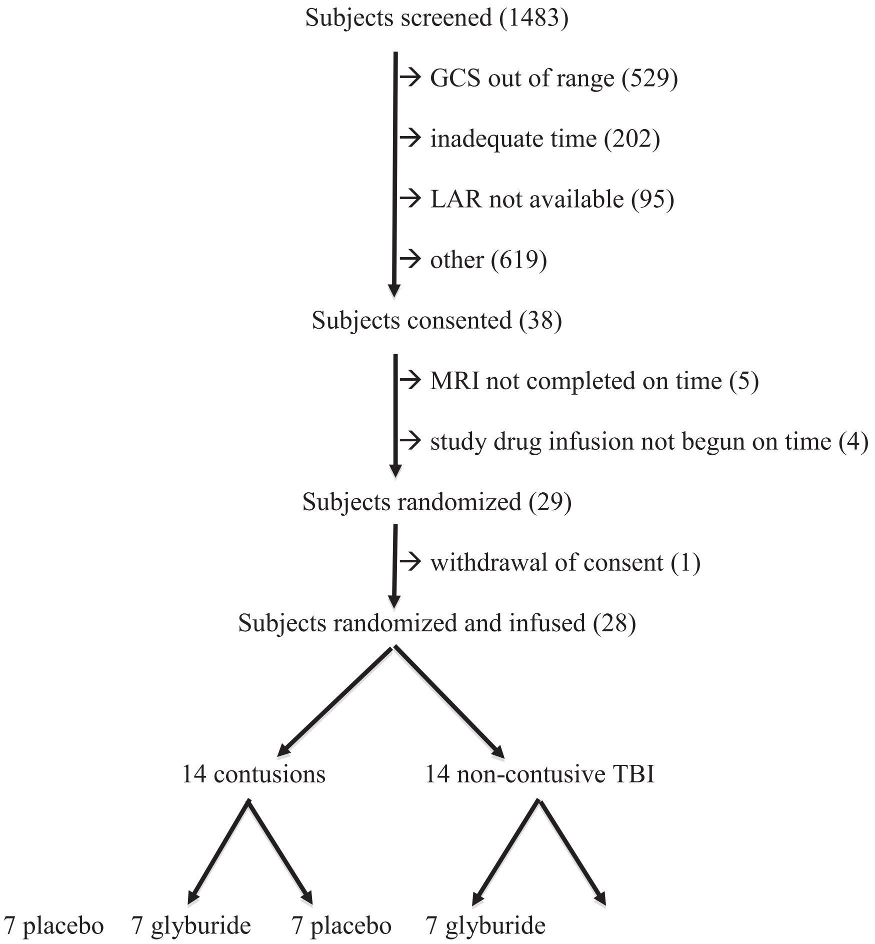

SUPPLEMENTARY FIG. S1. Patient enrollment. GCS, Glasgow Coma Scale; LAR, legally authorized representative; MRI, magnetic resonance imaging.

Supplementary Appendix S1. Inclusion/Exclusion Criteria

Inclusion criteria:

1. Documented closed-head traumatic brain injury (TBI)
2. Clearly defined time of injury no more than 10 h before administration of study drug/placebo
3. Glasgow Coma Scale (GCS) 4–14. The GCS will be obtained free of the effects of sedating and/or paralytic drug. Complicated mild must have GCS 13–14 and one or more of the following: intraparenchymal clots or contusions in aggregate >10 cc; midline shift >5 mm; IVH, SDH, EDH seen on more than one computed tomography (CT) scan slice.
4. Age 18–75 years
5. Patients in whom a dedicated peripheral intravenous (IV) line can be placed for study drug administration
6. Written consent obtained from legally authorized representative (LAR)

Exclusion criteria:

1. No documented TBI or time of impact not certain
2. Penetrating brain injury
3. Spinal column instability and/or spinal cord injury with neurodeficit
4. Concomitant severe non-survivable injury
5. Pregnant, or a positive pregnancy test
6. Women who intend to breastfeed during study days 1–4
7. Blood glucose <50 mg/dL
8. Severe renal disorder from the patient's history (e.g., dialysis) or serum creatinine of >2.5 mg/dL
9. Severe liver disease or total bilirubin >1.5 times upper limit of normal
10. International normalized ratio (INR) >1.4
11. Systolic blood pressure <90 mm Hg not responsive to fluid resuscitation
12. Blood alcohol >250 mg/dL

13. Inability to have magnetic resonance imaging (MRI) (pacemaker, non-MR compatible pressure monitor, etc.)
14. Hospitalization for brain injury, psychiatric or neurological disease within previous 3 years
15. Emergent or urgent surgical operation anticipated (in OR, bedside procedures excluded) that would prevent dosing with study drug within 8 h of injury
16. Known use of Coumadin (warfarin), Plavix (clopidogrel), Effient (prasugrel) or Pletal (cilostazol), heparin, low molecular weight heparin, heparinoids, or abciximab or similar antiplatelet agents in the previous 72 h (patients later found to have taken these medications will not be automatically excluded from the study)
17. Use of sulfonylurea drugs within the prior 30 days
18. Treatment with another investigational drug within the prior 30 days
19. Allergy to sulfonylurea drugs
20. Known diagnosis of G6PD enzyme deficiency
21. PaO_2 <60 mm Hg on admission (for patients in whom blood gases are drawn per standard of care)
22. Non-English speaking LAR and subjects (University of Maryland only)
23. Prisoners or others who may be unable to make a truly voluntary and uncoerced decision whether or not to participate in the study
24. Any other clinical condition that in the opinion of the investigator makes the patient unsuitable for inclusion into the study

Supplementary Appendix S2. Protocol for Managing Blood Glucose

Blood glucose (BG) monitoring and management

RP-1127 is a hypoglycemic agent. The dosing regimen used in this study is not expected to result in symptomatic hypoglycemia. Patients with traumatic brain injury (TBI) may be on insulin therapy. The combined effect of insulin and RP-1127 must be taken into account.

BG measurement. BG concentrations are to be measured by point-of-care (POC) testing of capillary blood, for example, using Accu-Chek. Alternately, provided it does not interfere with the timing specified below under "Monitoring," blood from an arterial line may be used if one is in place. Measurements of BG <70 mg/dL must be verified by laboratory test. The RP-1127 dosage must only be adjusted or stopped based on verified laboratory test values that are taken 15 min or more following the last administration of insulin. Treatment with intravenous (IV) dextrose will, however, not be withheld while waiting for laboratory test results; to avoid false readings, blood for the laboratory test must be drawn prior to treatment with IV dextrose.

Monitoring. BG monitoring will be hourly (± 15 min) for the first 24 h, every 2 h (± 30 min) for hours 24–48 and every 4 h (± 60 min) for hours 48–80. If BG <70 mg/dL, the hypoglycemia treatment protocol is initiated and BG monitoring is required every 15 min (± 10 min) until BG ≥80 mg/dL for three consecutive readings without exogenous glucose supplementation, then hourly for the next 12 h and every 2 h thereafter.

Hypoglycemia treatment. Any BG of <70 mg/dL will be treated with a bolus of D50W at a volume (mL) of (100 – BG in mg/dL) × 0.4. The table below, calculated using this formula, may be used as a guide.

Hypoglycemia treatment BG	*Volume of D50W to administer*
30 – 34	28
35 – 39	26
40 – 44	24
45 – 49	22
50 – 54	20
55 – 59	18
60 – 64	16
65 – 69	14

Insulin. If the patient is on insulin therapy, it must be stopped when BG <120 mg/dL or in accordance with the site's insulin therapy protocol if that protocol provides for a higher threshold, and may only be restarted when BG >150 mg/dL. Only IV insulin should be used, and "tight" BG control (80–110 mg/dL) is not permitted during administration of RP-1127.

Stopping rules. The RP-1127 dose must be reduced by 30% if: (i) there is 1 × lab-confirmed BG <40 mg/dL; (ii) there are 3 × lab-confirmed BG <70 mg/dL within a 12-h period; or (iii) lab-confirmed BG <55 mg/dL and the subject experiences a seizure that in the judgment of the investigator is related to hypoglycemia caused by RP-1127. The dose can only be reduced once; if any of (i) to (iii) above occurs a second time, RP-1127 must be discontinued. However, if the investigator determines that hypoglycemia was due to insulin error, the dose can be increased to the original starting dose, or restarted, as appropriate.

When RP-1127 is stopped for the reasons above, D50W should be administered by bolus to maintain BG >80 mg/dL. Multiple D50W boluses may be administered, the timing and volume of which are at the discretion of the clinician. BG monitoring is required every 15 (± 10) min until BG ≥80 for three consecutive readings without exogenous glucose supplementation, then hourly (±15 min) for the next 6 h. Every attempt should be made to follow this blood glucose monitoring and management protocol. However, the investigator has ultimate responsibility and will use his/her best judgment in treating any episodes of hypoglycemia based upon the specific clinical situation and in accordance with good practices. All instances of lab-confirmed hypoglycemia and treatment administered will be recorded in the Certified Research Forms.
